# Light Quality and Intensity Modulate Cold Acclimation in Arabidopsis

**DOI:** 10.3390/ijms22052736

**Published:** 2021-03-08

**Authors:** Sylva Prerostova, Petre I. Dobrev, Vojtech Knirsch, Jana Jarosova, Alena Gaudinova, Barbara Zupkova, Ilja T. Prášil, Tibor Janda, Břetislav Brzobohatý, Jan Skalák, Radomira Vankova

**Affiliations:** 1 Laboratory of Hormonal Regulations in Plants, Institute of Experimental Botany, Czech Academy of Sciences, Rozvojova 263, 16502 Prague, Czech Republic; dobrev@ueb.cas.cz (P.I.D.); knirsch@ueb.cas.cz (V.K.); jarosova@ueb.cas.cz (J.J.); gaudinova@ueb.cas.cz (A.G.); barbara.zupkova@gmail.com (B.Z.); vankova@ueb.cas.cz (R.V.); 2 Division of Genetics and Crop Breeding, Crop Research Institute, Drnovska 507, 16100 Prague, Czech Republic; prasil@vurv.cz; 3 Department of Plant Physiology, Agricultural Institute, Centre for Agricultural Research, ELKH, Brunszvik u. 2, 2462 Martonvásár, Hungary; janda.tibor@atk.hu; 4 Department of Molecular Biology and Radiobiology, Faculty of AgriSciences, Mendel University in Brno, Zemedelska 1, 61300 Brno, Czech Republic; brzoboha@ibp.cz (B.B.); jan.skalak@ceitec.muni.cz (J.S.); 5 CEITEC—Central European Institute of Technology and National Centre for Biomolecular Research, Masaryk University, Kamenice 5, 62500 Brno, Czech Republic

**Keywords:** auxin, combined stress, cryptochrome, cytokinin, gene expression, gibberellin, phytochrome, plant hormones

## Abstract

Plant survival in temperate zones requires efficient cold acclimation, which is strongly affected by light and temperature signal crosstalk, which converge in modulation of hormonal responses. Cold under low light conditions affected Arabidopsis responses predominantly in apices, possibly because energy supplies were too limited for requirements of these meristematic tissues, despite a relatively high steady-state quantum yield. Comparing cold responses at optimal light intensity and low light, we found activation of similar defence mechanisms—apart from *CBF1*–*3* and *CRF3*–*4* pathways, also transient stimulation of cytokinin type-A response regulators, accompanied by fast transient increase of *trans*-zeatin in roots. Upregulated expression of components of strigolactone (and karrikin) signalling pathway indicated involvement of these phytohormones in cold responses. Impaired response of *phyA*, *phyB*, *cry1* and *cry2* mutants reflected participation of these photoreceptors in acquiring freezing tolerance (especially cryptochrome CRY1 at optimal light intensity and phytochrome PHYA at low light). Efficient cold acclimation at optimal light was associated with upregulation of *trans*-zeatin in leaves and roots, while at low light, cytokinin (except *cis*-zeatin) content remained diminished. Cold stresses induced elevation of jasmonic acid and salicylic acid (in roots). Low light at optimal conditions resulted in strong suppression of cytokinins, jasmonic and salicylic acid.

## 1. Introduction

During evolution, plants have developed complex systems to sense environmental cues, transduce the information and regulate their growth and development to maximize their chances of survival and propagation [1]. Key cues include light signals, which provide spatial, temporal and seasonal information [2], not only in the intensity of photosynthetically active radiation (PAR), but also via specific wavelengths. Light signals are perceived by photoreceptors: red (R) and far-red (FR) light by phytochromes (in Arabidopsis: PHYA–E), and blue light by both cryptochromes (CRY1–3) and phototropins (PHOT1 and 2) [2].

The other important environmental cue is temperature. Plants may utilize this signal to regulate the timing of developmental transitions or to enhance tolerance to future temperature extremes [2]. Plants in temperate zones must cope not only with high but also low (including freezing) temperatures. Survival at sub-zero temperatures is usually enabled by efficient cold acclimation, a process regulated by both temperature and light signals. The reductions in ambient temperatures and daylengths in autumn accompanied by prolongation of twilight periods and reductions in R:FR ratio lead to plant cold acclimation (resulting in elevation of freezing tolerance) before onset of winter.

The light under low temperature conditions may induce photoinhibition, but simultaneously sufficient light intensity is required for effective cold acclimation [3,4,5]. Preservation of sufficient photosynthetic activity at low temperature seems to be essential for plants to acquire enough energy for induction of efficient stress defences (including expression of stress-related genes and synthesis of various protective compounds) and partial re-establishment of growth [4].

The R:FR ratio affects the activity of specific phytochromes in different ways. PHYB, the most abundant in above-ground tissues, is synthesized in an inactive form, activated by R and inactivated by FR. PHYA is activated by FR, exhibiting fast turnover [6,7,8]. PHYA mediates very low fluence and FR irradiance responses [9]. The phytochromes may be translocated from cytoplasm to the nucleus, where they interact, e.g., with phytochrome interacting factors (PIFs). PHYA activation by a combination of low R:FR ratio and temperature (4 °C) stimulates expression of C-repeat binding factors (CBFs) and, simultaneously induces accumulation of PIF4 in Arabidopsis [10]. At a low R:FR ratio, PHYA promotes *CBF* expression also at temperatures as high as 16 °C, e.g., in tomato [11]. The other phytochromes—PHYB and PHYD—are inactivated at low R:FR ratio.

CBFs were reported to stabilize PHYB, which contributes to elevation of freezing tolerance [12]. Light and temperature signals may converge on hormone signalling pathways. Sharabi-Schwager et al. [13] found that overexpression of *CBF2* upregulated transcription of 17 abscisic acid (ABA) biosynthetic and response genes in Arabidopsis. CBFs as well as transcription factor HY5, associated with light-induced photomorphogenesis and cold responses, upregulate expression of *GA2ox4*, which encodes a gibberellin-deactivating oxidase. Downregulation of gibberellins enhances the stability of DELLA proteins, which are important regulators involved in growth suppression, especially in multiple stress reactions [14]. HY5 is involved in feedback regulation of PHYA signal transduction [15]. HY5 can also activate ABA synthesis at low temperature by upregulating expression of *SlNCED6*, as well as ABA signalling by directly binding to the promoter of *ABI5* [10].

FR-activated PHYA is reportedly required for induction of ABA signalling and subsequent stimulation of jasmonic acid (JA) biosynthesis and signal transduction in cold conditions [11]. Hu et al. [16] found that JA enhances freezing tolerance through regulation of the ICE-CBF pathway in Arabidopsis. Thus, the CBF regulon-ABA-JA network is apparently connected in a self-amplifying cascade.

Salicylic acid (SA) is a simple phenolic compound that has long been recognized as a potent plant hormone [17], which participates in diverse signalling processes and plant defence through crosstalk with other hormones [18]. SA signalling leads to the reprogramming of gene expression and protein synthesis and may affect antioxidative responses and modulate cellular redox homeostasis [19,20].

Auxin role in cold stress responses has not been sufficiently elucidated. A low R:FR ratio was reported to promote auxin biosynthesis, but this effect has been predominantly studied at optimal temperature, as part of shade avoidance responses [21]. Auxin elevation has also been observed during high temperature acclimation [22].

Light exhibits an intensive crosstalk with the signalling pathway of plant hormones cytokinins (CKs). Type-A response regulator ARR4 modulates activity of PHYB, stabilizing its active form at low R:FR ratio [23]. Transient upregulation of the expression of other type-A ARRs, *ARR5*, *ARR6*, *ARR7* and *ARR15*, which are negative regulators of CK signal transduction, is an important part of early (maximum after 2 h) cold responses [24]. Fast attenuation of CK signal may be related to the necessity to suppress growth upon cold shock. However, exogenous CKs can enhance freezing tolerance [24], and levels of endogenous CKs increase during cold acclimation [25], clearly indicating that they play positive roles in later phases of plant cold stress responses related to acclimation to low temperatures. Our experiments with dexamethasone-inducible transformants with elevated and diminished CK content, respectively, showed that CKs positively affect plant cold tolerance [26]. CK effects do not seem to be related to the CBF signalling pathway [27].

As a follow-up of our previous study directed to the evaluation of the impact of CKs on the cold stress responses under different light conditions [26], the present study was aimed to elucidate the crosstalk between photoreceptors and hormonal signalling pathways in dependence on the light intensity during cold stress. Effects of light intensity and quality were examined by comparing reactions of wild-type (WT) Arabidopsis plants with phytochrome (*phyA* and *phyB*) and cryptochrome (*cry1* and *cry2*) mutants exposed to optimal and low light intensities under different temperature conditions. Hormonal responses were characterised in apices, leaves and roots after short- and long-term stress treatments. Changes in contents of active CKs, auxin, ABA, JA and SA were characterised and correlated with the expression of selected hormone-, stress- and photoreceptor-specific genes as well as with freezing tolerance, photosynthetic performance and membrane damage.

## 2. Results

The role of light in cold acclimation processes was evaluated by comparing *Arabidopsis thaliana* plants subjected to standard (S: 20 °C, 150 µmol m^−2^ s^−1^), low light (L: 20 °C, 20 µmol m^−2^ s^−1^), cold (C: 5 °C, 150 µmol m^−2^ s^−1^) or combination of cold and low light (CL: 5 °C, 20 µmol m^−2^ s^−1^) treatments for 7 d. The response of WT plants was compared with those of phytochrome (*phyA* and *phyB*) and cryptochrome (*cry1* and *cry2*) mutants. Under S, only *phyB* mutants showed clear phenotypic deviations from WT plants (resembling the shade avoidance syndrome): longer petioles, hyponasty and smaller leaf areas. Growth of all genotypes was reduced under L. Low temperature had a strong negative effect on growth of all genotypes under both C and CL (Figure S1).

### 2.1. Freezing Tolerance at Optimal and Low Light Intensity

**Figure 1:**
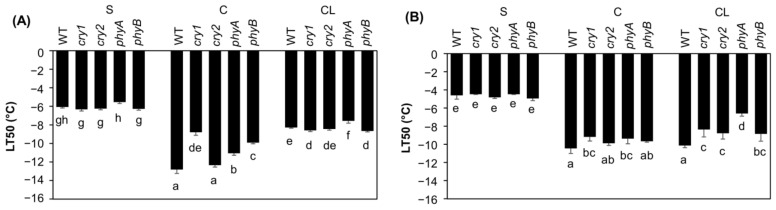
Freezing tolerance of (**A**) leaves and (**B**) whole plants expressed as LT50 values. Plants were pretreated by standard (S), cold (C) or combined cold and low light (CL) conditions for one week. Means ± SD are shown in plots. Between-treatment and between-genotype differences were evaluated by one-way ANOVA with Tukey’s test (*p* < 0.05, *n* = 6).

The control, non-acclimated plants showed the lowest freezing tolerance; no significant differences in survival rate were detected at standard light intensity among the tested genotypes. The LT50 values based on leaf damage measurements ranged from −6 °C (S) to −13 °C (C) (Figure 1A), and LT50 values based on plant survival rates ranged from −5 °C (S) to −10 °C (C) (Figure 1B). The differences between leaf damage and plant survival (LT50) indicate that most photoreceptors play an important role predominantly in leaves (under optimal light intensity). Following C, *cry1, phyB* and, to a minor extent *phyA*, exhibited lower tolerance than WT based on leaf damage. The most frost-sensitive genotype after cold acclimation under optimal light intensity, according to both leaf damage and plant survival, was *cry1*. Leaves subjected to cold stress had higher freezing tolerance under C than under CL (LT50: −13 °C and −8 °C, respectively, for WT leaves). Genotype *phyA* was the least frost-tolerant after the CL treatment. Only minor differences in leaf damage between WT and *cry1/phyB* under CL might be caused by the fact that at low light these photoreceptors are predominantly inactivated. Following the CL treatment, WT plants were the most tolerant in terms of survival rate.

### 2.2. Lipid Peroxidation

**Figure 2:**
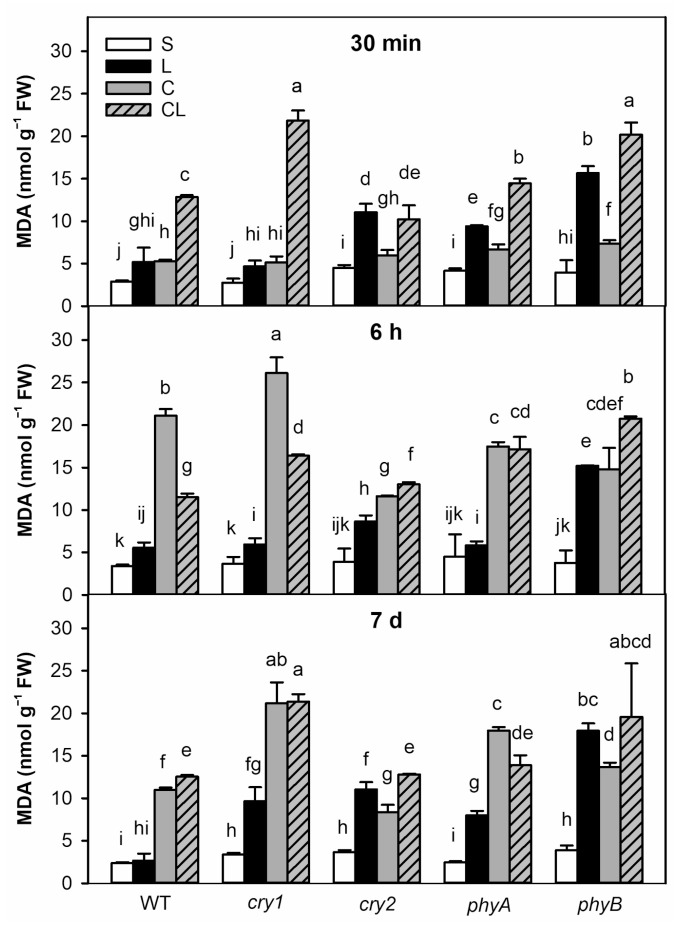
Membrane oxidative damage shown as malondialdehyde (MDA) levels in leaves of plants exposed to standard (S), low light (L), cold (C) and combined cold and low light (CL) conditions for 30 min, 6 h or 7 d. Means ± SD are shown in plots. Between-treatment and between-genotype differences were evaluated by one-way ANOVA with Tukey’s test (*p* < 0.05, *n* = 3). Statistical difference is indicated by different letters.

The malondialdehyde (MDA) level was used as a marker of membrane oxidative damage. Highly significant MDA elevation was found in *cry2* and, especially, *phyB* mutants after 6 h of the L treatment (Figure 2). After 7 d under L, all photoreceptor mutants exhibited enhanced lipid peroxidation, predominantly *phyB* mutant. In spite of reduced formation of reactive oxygen species due to the lower photosynthesis rate under L conditions, limited energy availability might result in decrease of the levels of reactive oxygen species scavengers and other protective compounds, leading to higher membrane damage. C caused substantial MDA increase in all genotypes; effects were strongest in *cry1* plants after 6 h and 7 d. WT was the only genotype able to diminish MDA content between 6 h and 7 d (but still not to level under S conditions). Under CL, MDA levels increased after 30 min. Since 6 h, the levels were similar to those observed in C-treated plants of all genotypes (except weaker response of WT and *cry1* plants after 6 h).

### 2.3. Effects of Photoreceptor Mutations on Photosynthetic Parameters

Effects of the specific photoreceptor mutations and cold treatment under each of the light conditions were characterised at the level of photosynthetic electron transport processes by measuring chlorophyll *a* fluorescence induction parameters after both short-term (6 h) and long-term (7 d) exposures (Table 1). No significant differences in F_v_/F_m_ [the maximum quantum efficiency of photosystem II (PSII)] were found between the genotypes under control conditions. L conditions first caused a significant transient increase in F_v_/F_m_, which reflects plant tendency to adapt to low light intensity. However, after 7 d this parameter tended to decrease.

Under C, decreases in F_v_/F_m_ were observed after 6 h in the WT plants (from 0.84 to 0.78) and the *cry2* mutant. F_v_/F_m_ was partly restored after 7 d under the C treatment, indicating that these growth conditions were not severely stressful and that plants activated acclimation mechanisms. Similarly, the photoinhibition induced by C was limited. The CL treatment caused a slight F_v_/F_m_ increase after 6 h, similarly as L. After 7 d, F_v_/F_m_ values were close to control ones.

Exposure to the L treatment for 6 h did not cause any reduction in actual quantum yield (QY__Lss_); in *phyB* mutant, even a slight increase was found. After 7 d, QY__Lss_ strongly decreased. The QY__Lss_ was slightly positively affected under C after 6 h, declining after 7 d. Under CL, QY__Lss_ was higher than in control conditions at both time points.

The most strongly affected parameter was non-photochemical quenching (NPQ__Lss_). While L caused only a small increase of NPQ__Lss_ (except the *phyA* mutant after 7 d), it fell in plants exposed to C for 6 h (most strongly in WT plants), staying partially reduced after 7 d. CL had a weaker effect than C.

### 2.4. Effects of Cold and Low Light Intensity on Hormonal Pools in WT Plants and Photoreceptor Mutants

#### 2.4.1. Cytokinins

**Figure 3:**
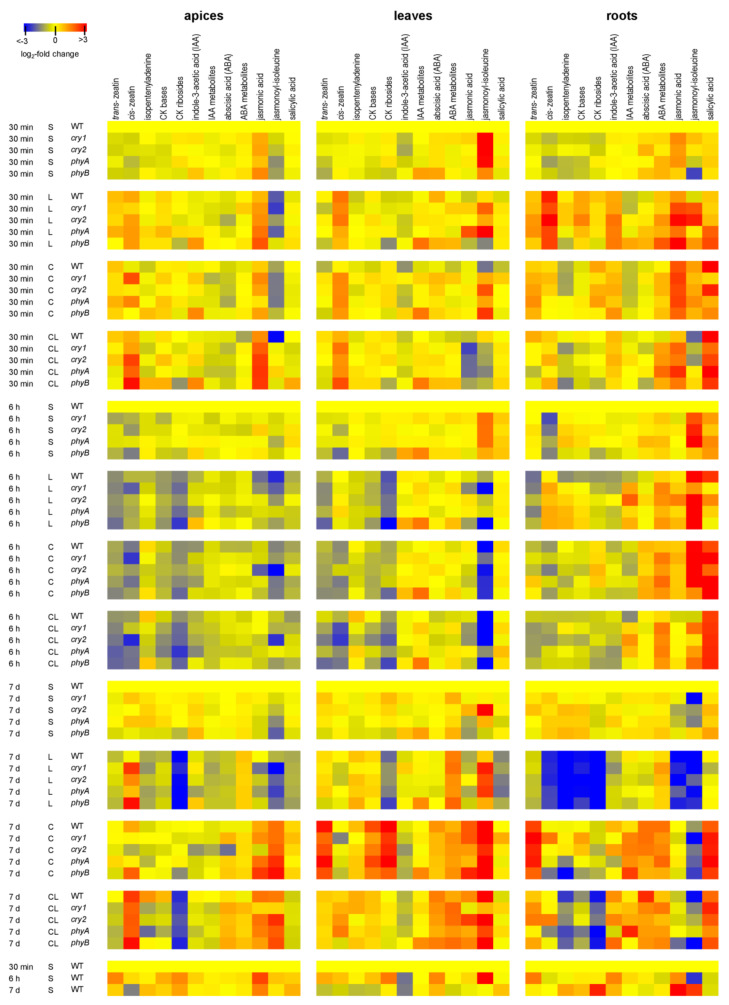
Heatmap of changes in phytohormone levels determined in shoot apices, leaves and roots of plants exposed to standard (S), low light (L), cold (C) and combined cold and low light (CL) conditions for 30 min, 6 h or 7 d. Four independent biological samples were evaluated. Data in the heatmap are normalised to WT levels in S conditions separately for each tissue and time point (the colour code shows relative changes in log_2_). Means ± SD and other statistics are shown in Table S2. Cytokinin (CK) bases (*trans*-zeatin, *cis*-zeatin, isopentenyladenine); CK ribosides (*trans*-zeatin riboside, dihydrozeatin riboside, *cis*-zeatin riboside, isopentenyladenosine); indole-3-acetic acid (IAA) metabolites (indole-3-acetic acid aspartate, indole-3-acetic acid glucosyl ester, oxo-indole-3-acetic acid, oxo-indole-3-acetic acid glucosyl ester); abscisic acid (ABA) metabolites (phaseic acid, dihydrophaseic acid, 9-hydroxy-abscisic acid).

Exposure to L for 6 h resulted in reduction in levels of active CK bases in WT plants and (to a lesser extent) the photoreceptor mutants (Figure 3). They reverted to control levels in leaves but were still suppressed in apices and roots after 7 d. The level of the most active CK *trans*-zeatin (*t*Z) began to rise after 30 min in apices and roots. Substantial *t*Z reductions were detected in leaves and roots of all genotypes after 6 h of the L treatment. After 7 d, *t*Z had returned to control levels in leaves.

Under the C treatment, moderate suppression of active CK bases was observed after 6 h in apices and leaves. After 7 d, CK bases, especially *t*Z, returned to control levels in apices and were substantially upregulated in leaves of all genotypes. In roots, noticeable elevation was observed after 7 d only in *cry1* plants, while the CK levels in the *cry2* mutant were downregulated after 6 h. In detail, elevated *t*Z content was found in roots of all genotypes after 30 min, and especially in leaves and roots after 7 d. The increase of *cis-*zeatin was found in leaves after 30 min, in apices only in *cry1*.

The combination of both stresses (CL) had negative effects on levels of CK bases after 6 h in all tissues, most strongly in *phyA* mutants. Strong downregulation of *t*Z, comparable to reductions observed under other stress treatments was detected. CK reduction, similar to C, was observed in leaves of all mutants; but after 7 d, levels of CK bases were comparable to those in controls. The CK basis decrease was observed in roots after 7 d, mostly in *phyA* mutants.

CK ribosides were downregulated after 6 h under L in all tested tissues of all genotypes but most strongly in apices (Figure 3, Table S2). Strong suppression was found especially after 7 d. Under C, levels of CK ribosides decreased in apices and leaves after 6 h. After 7 d, they were elevated in leaves of all genotypes. In roots, they were transiently enhanced in all genotypes (except *phyB*), generally returning to slightly less than control levels after 7 d. Under CL, CK riboside levels were decreased after 6 h in all tissues of all genotypes and even more after 7 d in apices and roots. CK precursors (CK phosphates; Table S2) were downregulated in all stress conditions in apices and leaves after 6 h. After 7 d, CK phosphates returned to control levels under C in apices, and their levels were enhanced in leaves. In roots, strong reductions of CK phosphates were detected after 7 d under L and (to a lesser degree) CL in all genotypes. In addition, levels of CK N-glucosides (*t*Z-type) were diminished under L and CL after 7 d in all genotypes (Table S2). In roots, CK N-glucosides were low in *phyA* plants under CL.

#### 2.4.2. Auxin

Indole-3-acetic acid (IAA) content was diminished in WT leaves after 30 min (especially under C), in apices after 6 h under all stress conditions (Figure 3). The response differed in *phyB,* which contained a higher basal level of IAA. This mutant maintained a higher level of IAA in apices and leaves after 30 min, later on especially under L. In roots, IAA was increased by L after 30 min. After 7 d, IAA suppression was detected in roots under L in all genotypes, while being upregulated under CL. The *phyA* mutant showed the strongest downregulation of IAA content under L and CL after 6 h, as well as under all conditions after 7 d. Under C, IAA levels were restored after 7 d only in WT apices and roots and in *cry2* roots. Levels of IAA metabolites were generally decreased after short-term C and CL treatments in apices, under L after 7 d (Figure 3). Their abundance was (similar to IAA) enhanced in leaves of *phyB*.

#### 2.4.3. Abscisic Acid

Moderate reductions in ABA levels were observed in WT apices under C treatment after 30 min and 6 h, after 7 d in all genotypes under L (Figure 3). The L treatment upregulated ABA only in WT leaves after 30 min. Under C and CL, ABA after 7 d increased in apices (except *cry2*) and especially in leaves and roots. Under C, the *cry2* mutant showed higher levels of ABA just after 6 h in apices, leaves and roots, but lower ones in apices after 7 d. Strong upregulation of ABA metabolites in all genotypes was found after 6 h of all stresses in roots and in leaves and roots after 7 d (Figure 3).

#### 2.4.4. Jasmonates

In apices, transient upregulation of JA was observed under CL after 30 min (Figure 3). After 6 h, moderate suppression was found under L (especially in WT), in *cry2* also under C. After 7 d, the JA levels were elevated under C and CL treatments in all genotypes except *cry2* under C. In leaves, early JA downregulation was detected under CL. Upregulation of JA was observed in *phyA* leaves under L. After 7 d, increase of JA levels was generally observed under C. In roots, all stresses resulted in JA elevation in all genotypes after 30 min, and mild elevation remained after 6 h under C. After 7 d, JA was strongly diminished in roots of all genotypes under L. Under C and CL, relatively higher content of JA was found in *phyA*, which was caused by low JA basal level at control conditions. Jasmonoyl-isoleucine (JA-Ile) trend was similar to that of JA after 7 d. In leaves, JA-Ile was transiently enhanced in *phyA* after 30 min under L. JA-Ile was diminished under C and CL after 30 min and 6 h in all genotypes. After 7 days it was upregulated under C and CL (except *cry2*). In roots, fast upregulation of JA-Ile was detected after 30 min under L in photoreceptor mutants and under C in all genotypes. After 6 h under C, JA-Ile levels were increased.

#### 2.4.5. Salicylic Acid

SA was generally slightly decreased in apices of all genotypes after 6 h (Figure 3). After 7 d, strong suppression was detected under L, almost none under CL and elevation under C in all genotypes. In leaves, SA downregulation was found under L after 7 d. Fast, strong positive responses to C and CL were detected in roots, which were maintained after 6 h and 7 d (*phyA* plants exhibited distinct responses under C and CL). Under L, transient increases were detected in *phy* mutants and later (after 6 h) in WT plants.

### 2.5. Transcription of Stress-Related Genes

**Figure 4:**
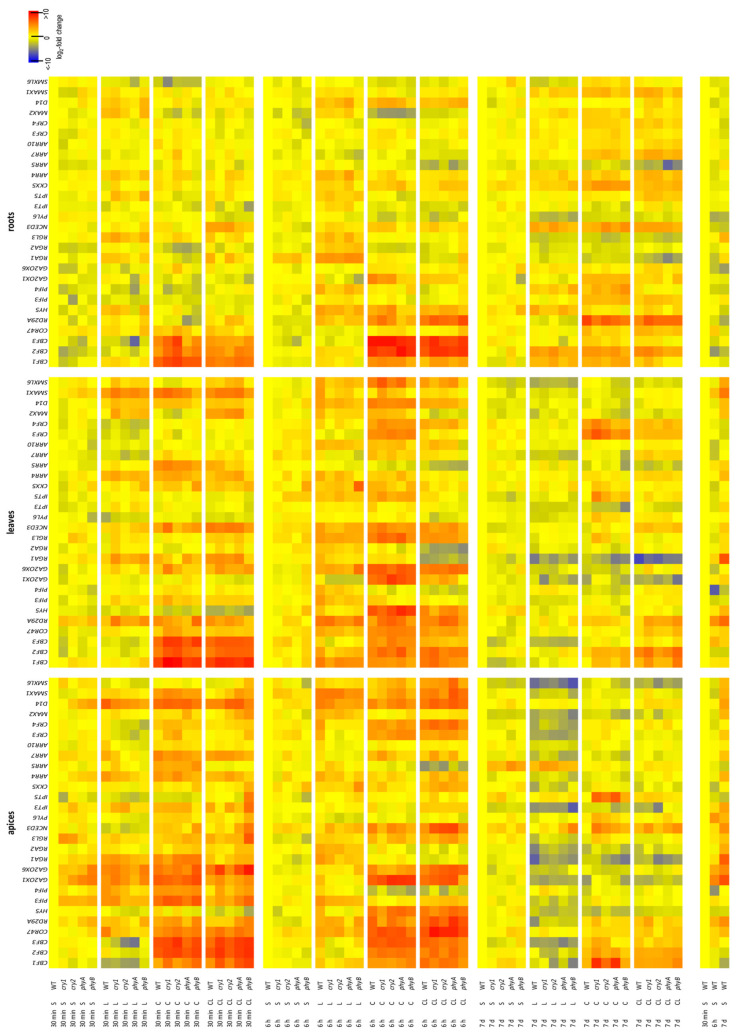
Heatmap of changes in transcript levels of selected stress-, phytohormone- and photoreceptor-specific genes. The expression was measured in shoot apices, leaves and roots of plants exposed to standard (S), low light (L), cold (C) and combined cold and low light (CL) conditions for 30 min, 6 h or 7 d. Four independent biological samples were evaluated. Data in heatmap are normalised to WT levels in S conditions separately for each tissue and time point. Means ± SD and other statistics are shown in Table S3.

The results of transcription analysis of stress- and hormone-related genes measured by RT-qPCR are shown in Figure 4 and Table S3.

#### 2.5.1. CBFs

One of the most important groups of cold-induced transcription factors are CBFs. Their expression was upregulated early (after 30 min) in all tissues under both C and CL, being elevated also after 6 h. In general, the response to C was stronger than in the case of CL. Only *CBF1* transcripts were elevated in apices under CL after 6 h. After 7 d, *CBFs* expression was close to control (except *cry1* and *phyA* apices under C). *CBF1* and *CBF2* were stimulated in leaves after 6 h and in roots after 6 h and 7 d, not only by C and CL but also by L. Relatively fast and higher expression was detected in *cry* mutants. *CBF1* and *CBF2* exhibited the highest expression after 30 min, but *CBF3* was more strongly expressed after 6 h.

#### 2.5.2. Other Stress-Inducible Genes

*COR47* expression was highly upregulated by C and CL treatments in all tissues of all genotypes after just 30 min and also by the L treatment in WT apices. After 6 h, very high stimulation was observed in apices, especially under CL, and under C in leaves. In leaves, *cry* mutants responded much more to C after 30 min than the other genotypes.

*RD29A* expression was moderately enhanced in apices of all genotypes after 30 min under C and in those of *phy* mutants also under CL. After 6 h and 7 d, *RD29A* expression was upregulated at C and CL. Strong responses in leaves were induced by all stresses after 6 h and after 7 d, mostly by CL. In roots, CL stimulated early responses in all genotypes except *cry1*. *RD29A* expression was low in *phy* mutants under C. Roots responded to CL and less strongly to C after 6 h as well as after 7 d (*phy* mutants responded later under C).

Expression of transcription factor *HY5*, which acts as a hub between light and CKs [1], was generally suppressed after 30 min of stresses in apices and leaves. It was strongly upregulated after 6 h under C and CL (less strongly in *cry1*). Suppression of *HY5* expression was evident in apices after 7 d of all stresses. In roots, *HY5* expression was stimulated under L (except in *phyA* mutant) after 30 min and under all stresses and genotypes after 6 h. Mild upregulation under L was also detected after 7 d.

Low light intensity, especially with low R:FR ratio, is associated with stimulation of expression of PIF transcription factors. Expression of *PIF3* strongly increased after 30 min under C in apices of all genotypes except *phyA* (in which the response was weaker), while *phyB* plants reacted also under CL. In leaves, all three stresses induced moderate increases in *PIF3* expression (especially in *cry1* plants). After 6 h, prominent responses to L were detected in apices and leaves (which were strongest by far in WT plants) and to CL in apices. In roots, moderate upregulation by C and CL was observed after 7 d. Expression responses of *PIF4* were similar to those of *PIF3*, but the responses to CL were earlier. Effects of L and CL partially remained in leaves after 7 d. *PIF4* expression stayed downregulated after 7 d only in WT apices. In roots, positive effects of the L treatment on *PIF4* were observed after 6 h and 7 d, as well as that of C after 7 d.

#### 2.5.3. Hormone-Related Genes

CBFs are known to stimulate expression of the gibberellin-deactivating enzymes GA2-oxidases [28]. Early upregulation of *GA2ox1* was observed in apices under C. After 6 h, strong increases in *GA2ox1* expression were observed in apices under C and to a slightly lesser extent under CL (except in *phyB* plants) but in leaves mostly under C. In roots, the abundance of *GA2ox1* transcripts increased after 6 h under C (less strongly in *phy* mutants than in other genotypes). *GA2ox6* was upregulated in apices by CL and C. In leaves, the stimulatory effect was stronger under C than under CL. Deactivation of gibberellins prevents degradation of DELLA proteins. Moreover, their expression was found to be promoted by the stresses. We detected transient increases in the abundance of *RGA1* and *RGA2* transcripts in apices and leaves under C and in all studied tissues under L and CL. *RGL3* expression was upregulated under C and CL in apices and leaves, as well as under L in all tissues. However, after 7 d, upregulation of *RGL3* expression was only detected in apices and leaves of *cry* mutants under C.

Steady stimulation of the gene encoding the rate-limiting ABA biosynthetic enzyme *NCED3* was found in apices and leaves under C and CL (under CL also in roots). Under L, *NCED3* expression was upregulated after 6 h in leaves and roots. After 7 d, *NCED3* was upregulated in roots by all stresses. One of the ABA receptor genes, *PYL6*, was negatively affected by L in apices and leaves after 30 min, in roots under C and CL after 6 h, and by all stresses after 7 d.

Expression of the CK biosynthetic gene *IPT3* was elevated in apices of all genotypes after 6 h, except *phyB* under C and *cry1* under CL. After 7 d, L as well as CL downregulated *IPT3* expression in apices, except of *phy* mutants under CL. *IPT3* expression was significantly suppressed in leaves after 7 d under all stresses (except in WT plants under CL) and upregulated after 6 h in roots under L. Expression of *IPT5* was highly upregulated after 30 min in apices of *phyB* plants under CL. Mild upregulation was found in leaves under C and L after 6 h and in roots under CL after 7 d. The CK oxidase/dehydrogenase gene *CKX5* was upregulated in apices under CL after 6 h (in *phyB* already after 30 min), in leaves under C and CL (after 30 min) and in roots under all stresses (after 6 h and 7 d).

Fast and strong upregulation of the type-A response regulator *ARR4* was observed in apices and leaves under all stresses within 6 h (under CL, the responses occurred after 6 h). In roots, moderate elevation was quite steady (except in *phyB* plants). *ARR5* was upregulated in apices and leaves under C after 30 min, but downregulated after 7 d under C and CL. Expression of the type-A response regulator *ARR7* was upregulated under C and CL in apices of all genotypes after 30 min and 6 h (especially in *cry1* plants). In roots upregulation was observed after 7 d under C and CL. The type-B response regulator *ARR10* was transiently induced under C in apices of all genotypes. *ARR10* expression was induced after 6 h by all stress treatments in leaves (except in *phyB* plants, which showed even suppression under CL) and in roots by C and CL after 7 d.

CK response factors (CRFs) also play important roles in abiotic stress responses. *CRF3* expression was rapidly stimulated in *cry* apices under C. After 6 h, strong upregulation under C and CL was detected in apices of all genotypes. *CRF3* expression was elevated under C in leaves (after 6 h and 7 d). *CRF4* was rapidly upregulated in apices under C and weakly under CL. Only WT plants responded significantly in this respect to L (similarly also *CRF3* gene). After 6 h, responses to C were maintained and responses to CL were considerably enhanced. In leaves, C induced a stronger response than CL, which was maintained from 6 h to 7 d. In roots, C and CL promoted *CRF4* expression after 7 d.

Strigolactones are plant hormones that play key roles in plant interactions with mycorrhizal fungi, but they are also involved in abiotic stress responses [29]. Expression of the strigolactone receptor gene *MAX2* was stimulated under L in apices after 6 h. In leaves, elevation was detected after 30 min under CL and L, as well as after 6 h under C and L, while in roots only L induced increases within 6 h (a delay was found in *phyA* mutant). High expression of *MAX2* was observed under all stresses after 7 d. The co-receptor α,β-hydrolase *D14* was strongly upregulated by all stresses in apices, leaves and roots within 6 h (in apices and leaves most strongly under CL and C, respectively). Expression of the strigolactone repressor *SMXL6* was rapidly stimulated under CL in *phyB* apices and moderately in all genotypes after 6 h. Strong suppression was found in apices after 7 d, especially under L and CL (except *cry* mutants under C). In leaves, rapid upregulation of *SMXL6* by L and CL was exceeded by response to C after 6 h (less in WT and *phyB* plants). In roots, negative effects of C were observed after 30 min and that of L after 6 h. As MAX2 is also a receptor in the karrikin pathway, expression of a repressor of this pathway, *SMAX1,* was also investigated and found to be upregulated after 30 min under C in apices and under all stresses in leaves. After 6 h, it was upregulated under all stress treatments in apices of all genotypes.

## 3. Discussion

The crosstalk between the photoreceptor-related signals and various hormonal interactions has been widely studied. However, the role of these photoreceptors in the cold acclimation processes, and the involvement of various hormones, especially CKs, are still poorly understood. In one of our previous works, we have shown that CKs positively affect plant cold tolerance [26]. Here, we demonstrate the role of CRY and PHY photoreceptors in the hormone-mediated light-dependent cold acclimation processes.

### 3.1. Low Light Intensity

**Figure 5:**
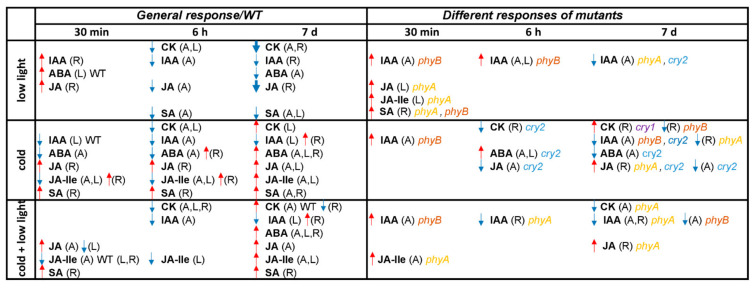
Scheme of significant changes in phytohormone contents. Upregulated (red arrows) or downregulated (blue arrows) hormone levels in shoot apices (A), leaves (L) and roots (R) under low light, cold at an optimal light intensity and combined cold and low light at different time points compared to control conditions. Left column contains either general response of all genotypes or WT (when specified). In the right column, responses of individual mutants distinct from the WT ones under the same stress conditions are shown. Thicker arrows indicate strong responses. ABA, abscisic acid; CK, cytokinin bases; IAA, indole-3-acetic acid; JA, jasmonic acid; JA-Ile, jasmonoyl-isoleucine; SA, salicylic acid.

Changes in light intensity affect activities of both phytochromes and cryptochromes. Low light intensity imposes in plants a shade avoidance response, especially in combination with a reduced R:FR ratio given by FR reflection and R absorption by neighbouring plants. Under low light, the most active photoreceptor is PHYA, which plays a crucial role in these conditions [9]. Deactivation of a large proportion of photoreceptors promotes the activation and stability of PIF transcription factors. Accordingly, upon transfer to L, expression of *PIF3* and *PIF4* was stimulated, especially in WT apices. Upregulation of *PIF* transcription factors (at optimal temperature) reportedly stimulates gibberellin, brassinosteroid and auxin biosynthesis (and auxin transport) [6]. We did not detect L-induced increases in auxin levels in apices, leaves or roots of most genotypes, IAA elevation was observed only in *phyB* apices and leaves (see Figure 5 for a summary of hormonal changes imposed by photoreceptor mutations). As PIFs have an important function in shade avoidance responses [27], upregulation of IAA levels in *phyB* plants indicates that PHYB knock-out strengthened the “shade” response, which is in accordance with the negative PHYB role in this process. The contrasting response of *phyA* plants in regulation of IAA content agrees with the positive function of PHYA in shade avoidance. The difference between WT and *cry2* responses suggests that decrease in blue light is sensed at L treatment, imposing an effect especially in the long term (Figure 5). PIF functions may be blocked by DELLA proteins [1]; thus, upregulation of *RGA1* and *RGA2* expression after 6-h L stress in all tissues suggests that *PIF3* and *PIF4* signals may be attenuated during prolonged stress.

Abiotic stress responses are generally associated with upregulation of ABA. At our sampling points (30 min and 6 h), no significant upregulation of ABA in any tissue was detected (except fast transient elevation in WT leaves). After 6 h, however, significant upregulation of expression of the ABA rate-limiting biosynthetic gene *NCED3* was found in leaves and roots, indicating possible subsequent ABA elevation. Under L, fast transient elevation of JA was observed only in leaves of *phyA* mutant (see Figure 5). After 6 h, suppression of JA levels was detected in apices, especially in WT plants. Downregulation of JA is consistent with reports of shade avoidance responses, including increases in plant susceptibility to biotic stresses due to reduction of JA signal transduction [30,31]. Contrasting reactions of *phyA* mutant (fast, strong increases of JA levels in leaves) confirms the importance of PHYA in L responses. In roots, significant JA reduction was detected after 7 d. SA levels were also diminished, particularly in apices and leaves. Transient elevation of SA levels in *phyB* apices and roots is consistent with reported negative effects of PHYB inactivation in the shade on SA biosynthesis [31].

CKs are plant hormones that intensively interact with light signalling. Transient upregulation of CK bases was detected in roots. After 6 h, levels of CK bases decreased in WT roots and after 7 d in roots of all genotypes. CK ribosides as well as CK precursors (CK phosphates) were strongly decreased after 6 h in all tissues. L was also associated with upregulation of expression of the negative CK response regulator *ARR4*, which was stimulated in all tissues, especially after 6 h. Upregulation of type-A ARRs may be important for attenuation of CK signal in this type of stress condition as well as for stabilization of PHYB [23].

We detected significant elevation of expression of strigolactone receptors *D14* and *MAX2* in apices, leaves and (most strongly) in roots, indicating that L responses are also associated with strigolactone signal transduction. The lack of *MAX2* response detected in *phyA* roots is in accordance with PHYA crucial role in L conditions. The expression pattern corresponds to the known organ-specificity of strigolactone biosynthesis, as roots are their primary production sites. This finding enhances knowledge of stress functions of these phytohormones, which reportedly participate in drought and salt responses [29].

### 3.2. Cold at Optimal Light Intensity

The integration of light signalling and cold stimuli is essential for induction of effective cold stress responses. In a study of photoreceptor roles, Wang et al. [14] found that *PHYA* expression was highly stimulated and *PHYB* expression suppressed after 6 h at 4 °C in tomato. PHYB was, however, found stabilized by CBFs in Arabidopsis in white light [12]. *CRY2* expression was reported to be cold induced in barley [32]. The effect of cold may be substantially strengthened by decreases in the R:FR ratio. Under these conditions, PHYA and PHYB were reported to be positive and negative regulators, respectively, of Arabidopsis ecotype La-er and tomato cold tolerance [11,33,34]. However, our tests on freezing tolerance of Arabidopsis Col-0 plants showed a different pattern in white light (Figure 1). WT plants had the highest survival rates and *cry1* plants the highest shoot frost sensitivity, which indicates that all followed photoreceptors participate in cold acclimation at optimal light intensity, especially blue light sensors. This is in accordance with Imai et al. [35], who reported a positive effect of blue light and cryptochromes on freezing tolerance in Arabidopsis. *phyA* and *phyB* plants had almost equal freezing tolerance, slightly lower than WT. This is consistent with findings by Kim et al. [36], who reported a positive effect of PHYB on CBF regulon and freezing tolerance. The differences in freezing tolerance observed in different studies might be at least partly due to differences in light spectra (as FR strongly enhances low temperature responses), plant species/cultivar or age.

Early responses to a temperature decrease included fast transient upregulation of *PIF3* and *PIF4* in apices and leaves (Figure 4). These factors interact with both phytochromes and cryptochromes [6]. PIFs are reportedly involved in regulation of expression of *CBF* transcription factors, both positive [14] and negative [12,37,38]. The strong upregulation of *PIF* expression, observed under C (Figure 4), indicates their important role in cold responses, distinct from shade avoidance. Similar *PIF* upregulation was reported after plant transfer from ambient to warm temperatures [39].

The most prominent transcription response was upregulation of *CBF1*–*3* expression (Figure 4). Our data are consistent with findings by Thomashow [40]—that *CBF* genes are induced within ca. 15 min of plant exposure to low temperature (4 °C). CBFs have positive effects on levels of DELLA proteins, which are strong growth suppressors involved especially in early phases of stress reactions, e.g., after cold shock. Accordingly, we found that *RGA1*, *RGA2* and *RGL3* were upregulated in apices and leaves under the C treatment. Apart from an enhanced abundance of DELLA proteins, gibberellin deactivation caused by *GA2ox1* and *GA2ox6* seems to be promoted, especially in apices. The involvement of these gibberellin deactivating enzymes in cold responses was first described by Achard et al. [28].

ABA and JA play crucial roles in cold stress responses. ABA stimulates expression of many protective genes, including dehydrins, an abundance of which positively correlates with the extent of freezing tolerance [41]. It also enables restoration of water potential after cold stress-induced reductions in root hydraulic conductivity, which impairs xylem water transport. We did not detect significant ABA peaks at the early time points in our analyses, possibly because they generally occur after 24 h of cold stress [25,42]. Nevertheless, *NCED3* upregulation was found in all tissues after 6 h. We also detected accumulation of ABA deactivation products in roots after 6 h and in all tissues after 7 d, indicating that the ABA peak preceded this sampling point. After 7d, ABA was slightly elevated, with the exception of *cry2* which exhibited strong downregulation in apices. Low levels of ABA and JA in *cry 2* apices after prolonged cold stress (Figure 5) indicate the importance of blue light in effective cold acclimation.

JA also promotes accumulation of cryoprotective compounds, especially polyamines, glutathione and anthocyanins [43]. After cold shock, DELLA proteins (especially RGA1) might promote JA signal transduction in apices and leaves by competitive binding to JA repressors—JAZ proteins [44]. Suppression of JAZ function may release inhibition of ICE1, an activator of CBF expression [43]. Stimulation of the CBF signalling pathway seems to be associated with early upregulation of JA in roots (Figure 3). JA levels were upregulated in apices and leaves after 7 d acclimation (except in *cry2* apices, which maintained the same JA levels during the whole experiment), in accordance with previous reports [25].

In contrast to the positive reported impact of exogenous SA on cold tolerance in various plant species [45,46], we did not find significant effect of our C treatments on endogenous SA levels in apices or leaves until 7 d (Figure 3). However, SA levels were already upregulated in roots of all genotypes after 6 h. This finding is consistent with observations by Dong et al. [47] of a rapid SA increase in cucumber roots and a 3 d delay in shoots. Similarly, Kim et al. [48] found SA elevation during the second week of cold treatment in Arabidopsis plants.

The other prominent cold stress response pathways, generally considered independent of CBFs [27], include CK type-A response regulators (specifically ARR5–7, ARR15) [24] and CRFs (especially CRF2–4) [49,50]. Accordingly, *ARR4, ARR5* and *ARR7* expression was rapidly upregulated by our C treatments, especially in apices, while *CRF3* and *CRF4* transcript abundance was elevated in apices and leaves, especially after 6 h (Figure 4). The stimulation of *ARR* expression requires functional CK receptors AHK2 and AHK3, phosphotransfer proteins (AHP2, AHP3, and AHP5) and type-B ARR1 [24,51]. Despite downregulation of active CKs in directly exposed apices and leaves (especially in WT plants), an early transient increase of *t*Z was observed in roots. This elevation may represent a short-term, xylem-based signal, inducing type-A ARR transcription in apices and leaves, which also exhibits a transient expression profile (maximum after 1–4 h [24]). Transient stimulation of CK signal transduction (within 7 min) was also observed in the non-exposed roots after heat stress targeted to shoots [52].

In contrast to L responses, levels of CK bases and ribosides increased after 7 d C in leaves and in the case of tZ also in roots of all genotypes, indicating their ability to acclimate to low temperature conditions (at sufficient light intensity) and restore growth, at least partially. This conclusion is in accordance with our data on positive effects of CKs on cold stress tolerance demonstrated using Arabidopsis overexpressing *ipt* gene driven by dexamethasone-inducible promoter [26]. The levels of the other growth-promoting hormone, auxin, were repressed by C, being restored predominantly in WT apices and roots after 7 d. Substantially lower levels of IAA were detected in *cry2* and *phyB* apices (Figure 5). Cold-induced downregulation of auxin levels was reported to be mediated by CK signalling [53].

The transcription factor HY5 represents a point of convergence between CKs and blue light signals [54]. In contrast to L responses, C was associated with *HY5* promotion in apices, leaves and (to a lesser extent) roots after 6 h. The diminished stimulation of *HY5* in *cry1* apices is consistent with its participation in blue light signal transduction. Mild stimulation of *HY5* expression in leaves after 7 d (except in *phyB* plants) coincided with increases in CK contents, in accordance with reported positive effects of CKs on HY5 stability [54].

The detected upregulation of expression of the strigolactone receptor *D14* in all tissues in response to cold treatments, together with elevation of the receptor *MAX2*, indicate potential involvement of strigolactone and karrikin pathways. Early increases in levels of the repressor of the karrikin pathway in apices and leaves, with subsequent elevation of the strigolactone repressor, suggest that both pathways may be involved in cold stress responses. This is consistent with strigolactone function in other abiotic stress responses [29].

Enhanced frost sensitivity of *cry1* plants exposed to C (Figure 1) was associated with elevation of expression of *ARR7* in apices and *PIF3* in leaves. After 7 d, *cry1* plants maintained upregulation of components of protective pathways: *CBF1* and *RGL3* in apices, *RGL3*, *CRF3*, *CRF4* in leaves, as well as stress hormone-related genes *NCED3* and *PYL6* in apices and *SMXL6* in leaves. All photoreceptors seem to be involved in downregulation of *PIF4* in apices after 7 d.

### 3.3. Cold at Low Light Intensity

In order to pinpoint changes in cold responses associated with insufficient light intensity, we compared responses to C and CL. CL led to enhanced expression of *CBF*s and cold-inducible genes in apices (Figure 4). Similarly, *CRF4* expression was substantially elevated in apices after 6 h. In addition, fast JA upregulation in apices was generally slightly higher under CL than under C (Figure 3). Promotion of defence indicates that these meristematic tissues may have stronger stress sensitivity under CL. Plant stress responses include activation of defence as well as regulation of plant development. Upregulation of the strigolactone pathway, which has a negative effect on shoot growth, together with downregulation of CK biosynthesis (reflected by suppression of CK phosphates and ribosides and upregulated *CKX5* expression), indicate that reduction in PAR strongly affects shoot apical meristems. Our data are consistent with conclusions by van der Schoot and Rinne [55]—that cold may primarily affect shoot apical meristems. The *PIF* elevation was weaker in apices under CL than under C. Faster *PIF* dynamics in both cold treatments in comparison with L indicates specific responses at low temperature. In contrast, expression dynamics of *ARR7* and *RGL3* under CL did not differ from those observed under C.

In mature leaves, relatively high QY__Lss_ detected after 7 days and low NPQ__Lss_ detected after 6 h and 7 days indicate adaptability to low PAR (Table 1)**.** Nevertheless, after 7 d, while cold acclimation associated with upregulation of CKs in leaves and roots occurred under C, the CL-treated plants exhibited substantially lower active CK levels (except cZ in apices) as well as lower freezing tolerance (Figs. 1, 3), which is in accordance with Janda et al. [56]. Levels of CK ribosides and phosphates were substantially decreased. The CL treatment led to upregulated expression of type-A *ARRs* (Figure 4). ABA levels (and *NCED3* expression) were generally higher after 7 d under CL than under C. CL positively affected *PIF4* expression in leaves. In contrast, expression of the DELLA protein *RGA1* after 7 d was lower in leaves and roots under CL than under C.

The crucial role of PHYA in responses to low light fluence was reflected by substantially decreased freezing tolerance of the *phyA* mutant under CL (Figure 1). The greater sensitivity of *phyA* plants was indicated by stronger *RD29A* upregulation in apices after 6 h (Figure 4). The impaired acclimation of *phyA* plants may be related to lower levels of CK bases (as well as CK N-glucosides) and SA in roots after 7 d. Comparison of CL and L responses allowed to evaluate the contribution of the “shade” response via disturbed responses of *phy* mutants—enhanced IAA levels in *phyB* apices or decreased IAA levels (together with increased JA and JA-Ile levels) in *phyA* apices and roots.

### 3.4. Conclusion

The summary of phytohormone changes induced by individual stress treatments is shown in Figure 5. The obtained data clearly illustrate that complex crosstalk between light and temperature signals involves at optimal light intensity all tested photoreceptors (especially cryptochromes), while at low light predominantly PHYA. The signal transduction is at least partly mediated by phytohormones. Cold acclimation at optimal light intensity was associated with upregulation of *trans*-zeatin in leaves and roots, while the combination of cold and low light was associated with an increase of *cis-*zeatin in apices. Both cold stress treatments induced elevation of JA and SA (in roots).

## 4. Materials and Methods

### 4.1. Plant Material and Experimental Conditions

To study effects of light quality on cold acclimation, the following *Arabidopsis thaliana* (L.) ecotype Col-0 mutants were obtained from the Arabidopsis Biological Resource Center: *phyA* (SALK_014575), *phyB* (SALK_022035), *cry1* (SALK_042397C) and *cry2* (SAIL_763_D08). WT plants were used as controls. Seeds were vernalized in water for 2 days (4 °C, dark) and cultivated for 4 weeks in Araponics hydroponics system (Liège, Belgium; 1.7 l tank; 1/4 Hoagland solution) in a climate chamber, Percival AR41-L2 (Percival Scientific, Perry, IA, USA; for the light spectrum see Figure S2), providing 8/16 h light/dark photoperiod, with optimal light intensity 150 μmol m^−2^ s^−1^, 20 °C temperature and ca. 60% relative humidity. A short day regime was chosen as it promotes cold stress responses and ensures that all genotypes remain in a vegetative state. The medium was aerated every 3 h and changed after 3 weeks of cultivation. The medium of the 4-week-old plants was changed again and they were cultivated for a further week in standard conditions (S: 20 °C, 150 µmol m^−2^ s^−1^) or exposed to the following stresses: low light conditions (L: 20 °C, 20 µmol m^−2^ s^−1^), cold (C: 5 °C, 150 µmol m^−2^ s^−1^, precooled medium) or combination of both cold and low light (CL: 5 °C, 20 µmol m^−2^ s^−1^, precooled medium). As cold and light have interactive effects, these treatments were started at the beginning of a light period. Potential effects of circadian rhythms were minimized by comparing stress-treated samples with controls at the same time points. Independent biological samples were collected after 30 min, 6 h and 7 days (d) of each treatment.

The experiment was repeated three times. Samples for MDA, phytohormone and qPCR analyses were frozen in liquid nitrogen and stored at −80 °C.

### 4.2. Freezing Tolerance

The freezing tolerance of sets of 5–10 plants of each genotype subjected to S, L and CL was tested (with three repetitions) by placing them in plastic holders in seven freezers under controlled temperature conditions [57]. After gradually decreasing the temperature to −2 °C, the plants were sprinkled with small ice crystals and left for 4 h to ensure extracellular freezing. Temperatures in the freezers were then reduced by 2 °C h^−1^ to −4, −6, −8, −10, −12, −14 or −16 °C and these temperatures were held for 12 h. After gradually warming the plants back to room temperature (2 °C h^−1^), the extent of frost damage in half of the samples was evaluated by conductometric measurement of membrane leakage in the leaves [57]. The remaining plants were used to assess survival rates after 3 weeks regeneration in nutrient solution at 20 °C with a 16 h photoperiod and optimal light intensity. Following Janáček and Prášil [58], freezing tolerance was expressed in terms of LT50: the temperature at which 50% of the leaves were damaged or 50% of the plants did not survive.

### 4.3. Lipid Peroxidation

Lipid peroxidation was evaluated in leaves by measuring their malondialdehyde (MDA) contents using the thiobarbituric acid-reactive-substances (TBARS) method, with spectrophotometric determination of the reaction products at 532 nm and taking into account the absorbance of interfering compounds following Hodges et al. [59] and Landi [60].

### 4.4. Chlorophyll Fluorescence Measurement

Six mature leaves of plants sampled after 6 h and 7 d of the treatments were cut and dark-adapted for 15 min at the same temperature as their respective treatments. They were then subjected to fluorescence measurements with Handy FluorCam FC 1000-H (Photon Systems Instruments, Drasov, Czech Republic) in Pulse-Amplitude-Modulated Mode. Chlorophyll florescence was measured during dark adaptation phase (duration 5 s) followed by saturating pulse (2000 µmol m^−2^ s^−1^; 800 ms), dark relaxation (40 s) and actinic light period (200 µmol m^−2^ s^−1^, duration 10 min) with 15 saturating flashes. The maximum photosystem II (PSII) quantum yield in the dark-adapted state (F_v_/F_m_) and steady-state PSII quantum yield in the light (QY__Lss_) were calculated according to Genty et al. [61]. Steady-state non-photochemical quenching in the light (NPQ__Lss_) was measured according to Horton and Ruban [62].

### 4.5. Phytohormone Analysis

Samples (ca. 10 mg FW) of shoot apical meristem with the four youngest leaf primordia (apex), developed leaves and roots were homogenized and extracted with 100 µL 50% acetonitrile solution. The following isotope-labelled standards were added at 1 pmol per sample: ^13^C_6_-IAA (Cambridge Isotope Laboratories, Tewksbury, MA, USA); ^2^H_4_-SA (Sigma-Aldrich, St. Louis, MO, USA); ^2^H_3_-PA, ^2^H_3_-DPA (NRC-PBI); ^2^H_6_-ABA, ^2^H_5_-JA, ^2^H_5_-*t*Z, ^2^H_5_-*t*ZR, ^2^H_5_-*t*ZRMP, ^2^H_5_-*t*Z7G, ^2^H_5_-*t*Z9G, ^2^H_5_-*t*ZOG, ^2^H_5_-*t*ZROG, ^15^N_4_-*c*Z, ^2^H_3_-DZ, ^2^H_3_-DZR, ^2^H_3_-DZ9G, ^2^H_3_-DZRMP, ^2^H_7_-DZOG, ^2^H_6_-iP, ^2^H_6_-iPR, ^2^H_6_-iP7G, ^2^H_6_-iP9G, ^2^H_6_-iPRMP ^2^H_2_-GA_19_, (^2^H_5_)(^15^N_1_)-IAA-Asp and (^2^H_5_)(^15^N_1_)-IAA-Glu (Olchemim, Olomouc, Czech Republic). The extracts were centrifuged at 4 °C and 30,000× *g*. The supernatants were applied to SPE Oasis HLB 96-well column plates (10 mg/well; Waters, Milford, MA, USA) activated with 100 µL methanol and then eluted with 100 µL 50% acetonitrile using Pressure+ 96 manifold (Biotage, Uppsala, Sweden). The pellets were re-extracted in 100 µL portions of 50% acetonitrile, centrifuged and applied again to the column plates. Phytohormones in each eluate were separated on Kinetex EVO C_18_ column (2.6 µm, 150 × 2.1 mm, Phenomenex, Torrance, CA, USA). Mobile phases consisted of A—5 mM ammonium acetate and 2 µM medronic acid in water and B—95:5 acetonitrile:water (*v*/*v*). The following gradient was applied: 5% B in 0 min, 5–7% B (0.1–5 min), 10–35% B (5.1–12 min) and 35–100% B (12–13 min), followed by a 1 min hold at 100% B (13–14 min) and return to 5% B. Hormone analysis was performed with a LC/MS system consisting of UHPLC 1290 Infinity II (Agilent, Santa Clara, CA, USA) coupled to 6495 Triple Quadrupole Mass Spectrometer (Agilent, Santa Clara, CA, USA), operating in MRM mode, with quantification by the isotope dilution method. Data acquisition and processing was performed with Mass Hunter software B.08 (Agilent, Santa Clara, CA, USA).

### 4.6. Gene Expression Quantification

Samples from apices, leaves and roots (ca. 100 mg samples) were homogenized with zirconia balls in a cooled ball mill MM301 (Retsch, Haan, Germany) for 150 s at 25 Hz. RNA was isolated using RNeasy Plant Mini Kit (Qiagen, Hilden, Germany) and treated with DNase I recombinant (Roche Applied Science, Penzberg, Germany) following the manual. Total mRNA was translated to cDNA using M-MLV Reverse Transcriptase (RNase-H Minus, Point Mutant, Promega, Madison, WI, USA), oligo-dT primers and Protector RNase Inhibitor (Roche Applied Science, Penzberg, Germany). Final cDNA (20-times diluted) was mixed with 5 μL GoTaq qPCR Master Mix (Promega, Madison, WI, USA) and specific primers (see Table S4) to a final volume of 10 μL. Target sequences were amplified by PCR, with cycles of 10 s at 95 °C for primer denaturation and 30 s at 60 °C for annealing and elongation, using Light Cycler 480 (Roche Applied Science, Penzberg, Germany). Ubiquitin UBQ10 was selected as the reference gene as it was stably transcribed in all the genotypes and tissues under all the treatments and time points, in accordance with the Genevestigator database [63]. Relative RNA contents were calculated by the ddCt method [64].

### 4.7. Statistics

Differences in measured variables related to the treatments and genotypes were analysed by one-way ANOVA with Tukey’s post hoc test (*p* < 0.05) using Prism 8 (GraphPad, San Diego, CA, USA). Numbers of independent biological repetitions in specific analyses are mentioned above.

## Figures and Tables

**Table 1:** Photosynthetic parameters: the maximum quantum yield of photosystem II (PSII) in the dark-adapted state (F_v_/F_m_), steady-state quantum yield (QY*__Lss_*) and non-photochemical quenching (NPQ*__Lss_*) of PSII. Selected genotypes were exposed to standard (S), low light (L), cold (C) and combined cold and low light (CL) conditions for 6 h or 7 d. Means ± SD of six independent developed leaves are presented. WT, wild type. Between-treatment and between-genotype differences were evaluated by one-way ANOVA with Tukey’s test (*p* < 0.05, *n* = 6)—see Table S1.

			S	L	C	CL
***Fv/Fm***	**6 h**	**WT**	0.838 ± 0.007	0.853 ± 0.004	0.783 ± 0.005	0.847 ± 0.005
		***cry1***	0.822 ± 0.019	0.844 ± 0.007	0.802 ± 0.012	0.847 ± 0.007
		***cry2***	0.835 ± 0.008	0.850 ± 0.001	0.810 ± 0.008	0.850 ± 0.006
		***phyA***	0.835 ± 0.005	0.856 ± 0.006	0.821 ± 0.016	0.850 ± 0.006
		***phyB***	0.837 ± 0.011	0.859 ± 0.003	0.827 ± 0.005	0.852 ± 0.009
	**7 d**	**WT**	0.846 ± 0.005	0.799 ± 0.014	0.810 ± 0.001	0.848 ± 0.004
		***cry1***	0.840 ± 0.005	0.809 ± 0.014	0.797 ± 0.005	0.858 ± 0.004
		***cry2***	0.849 ± 0.003	0.800 ± 0.007	0.817 ± 0.005	0.852 ± 0.004
		***phyA***	0.841 ± 0.010	0.802 ± 0.009	0.809 ± 0.015	0.850 ± 0.006
		***phyB***	0.851 ± 0.003	0.828 ± 0.009	0.814 ± 0.007	0.856 ± 0.005
***QY__Lss_***	**6 h**	**WT**	0.540 ± 0.027	0.544 ± 0.024	0.547 ± 0.017	0.555 ± 0.050
		***cry1***	0.517 ± 0.019	0.523 ± 0.032	0.554 ± 0.014	0.597 ± 0.007
		***cry2***	0.543 ± 0.022	0.548 ± 0.013	0.573 ± 0.014	0.610 ± 0.001
		***phyA***	0.575 ± 0.017	0.565 ± 0.015	0.583 ± 0.023	0.612 ± 0.007
		***phyB***	0.512 ± 0.033	0.561 ± 0.041	0.577 ± 0.012	0.652 ± 0.057
	**7 d**	**WT**	0.576 ± 0.017	0.405 ± 0.023	0.520 ± 0.008	0.633 ± 0.005
		***cry1***	0.544 ± 0.010	0.381 ± 0.021	0.530 ± 0.008	0.637 ± 0.005
		***cry2***	0.558 ± 0.016	0.424 ± 0.052	0.537 ± 0.033	0.642 ± 0.007
		***phyA***	0.577 ± 0.024	0.482 ± 0.013	0.573 ± 0.022	0.630 ± 0.001
		***phyB***	0.589 ± 0.020	0.498 ± 0.035	0.526 ± 0.026	0.610 ± 0.028
***NPQ__Lss_***	**6 h**	**WT**	1.093 ± 0.071	1.118 ± 0.090	0.310 ± 0.028	0.807 ± 0.112
		***cry1***	0.950 ± 0.114	1.007 ± 0.120	0.528 ± 0.028	0.619 ± 0.051
		***cry2***	0.955 ± 0.101	1.020 ± 0.060	0.477 ± 0.038	0.630 ± 0.050
		***phyA***	0.882 ± 0.070	1.040 ± 0.102	0.575 ± 0.118	0.653 ± 0.023
		***phyB***	1.118 ± 0.117	1.120 ± 0.128	0.743 ± 0.057	0.618 ± 0.153
	**7 d**	**WT**	1.002 ± 0.056	1.298 ± 0.106	0.697 ± 0.039	0.687 ± 0.039
		***cry1***	1.173 ± 0.144	1.417 ± 0.114	0.547 ± 0.069	0.663 ± 0.039
		***cry2***	1.036 ± 0.094	1.196 ± 0.196	0.684 ± 0.113	0.660 ± 0.026
		***phyA***	0.916 ± 0.139	0.880 ± 0.240	0.653 ± 0.078	0.777 ± 0.086
		***phyB***	0.967 ± 0.090	0.957 ± 0.158	0.641 ± 0.069	0.784 ± 0.102

## Data Availability

The data presented in this study are available in this article and supplementary material.

## Supplementary Materials

The following are available online at https://www.mdpi.com/1422-0067/22/5/2736/s1.
