# Supplementary material for: Light Quality and Intensity Modulate Cold Acclimation in Arabidopsis
Source: Int J Mol Sci. 2021 Mar 8;22(5):2736. doi: 10.3390/ijms22052736 (PMC7962961; doi:10.3390/ijms22052736)
Supplement: Supplementary file 1 [file ijms-22-02736-s001.zip › Figure S1-plant phenotype.pdf]

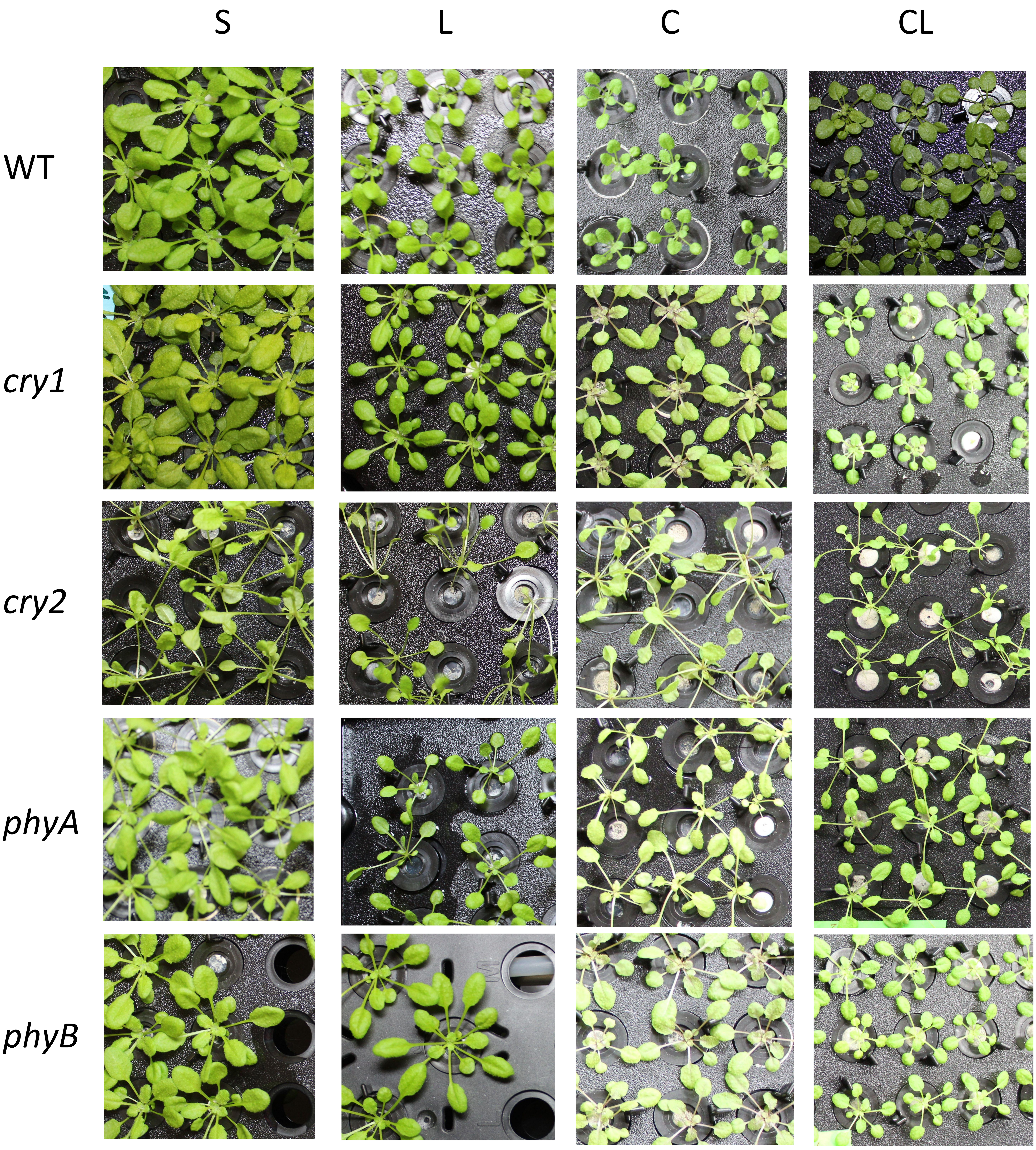


**Figure S1:** Phenotype of the five tested genotypes (WT, *cry1* , *cry2* , *phyA* , and *phyB* ) exposed to experimental conditions for 7 days. S - standard conditions (20°C, 150 µmol $m^{-2}$ $s^{-1}$); L - low light conditions (20°C, 20 µmol $m^{-2}$ $s^{-1}$); C - cold (5°C, 150 µmol $m^{-2}$ $s^{-1}$); CL - cold under low light conditions (5°C, 20 µmol $m^{-2}$ $s^{-1}$).
