# Supplementary material for: Light Quality and Intensity Modulate Cold Acclimation in Arabidopsis
Source: Int J Mol Sci. 2021 Mar 8;22(5):2736. doi: 10.3390/ijms22052736 (PMC7962961; doi:10.3390/ijms22052736)
Supplement: Supplementary file 1 [file ijms-22-02736-s001.zip › Figure S2-Light spectrum.pdf]

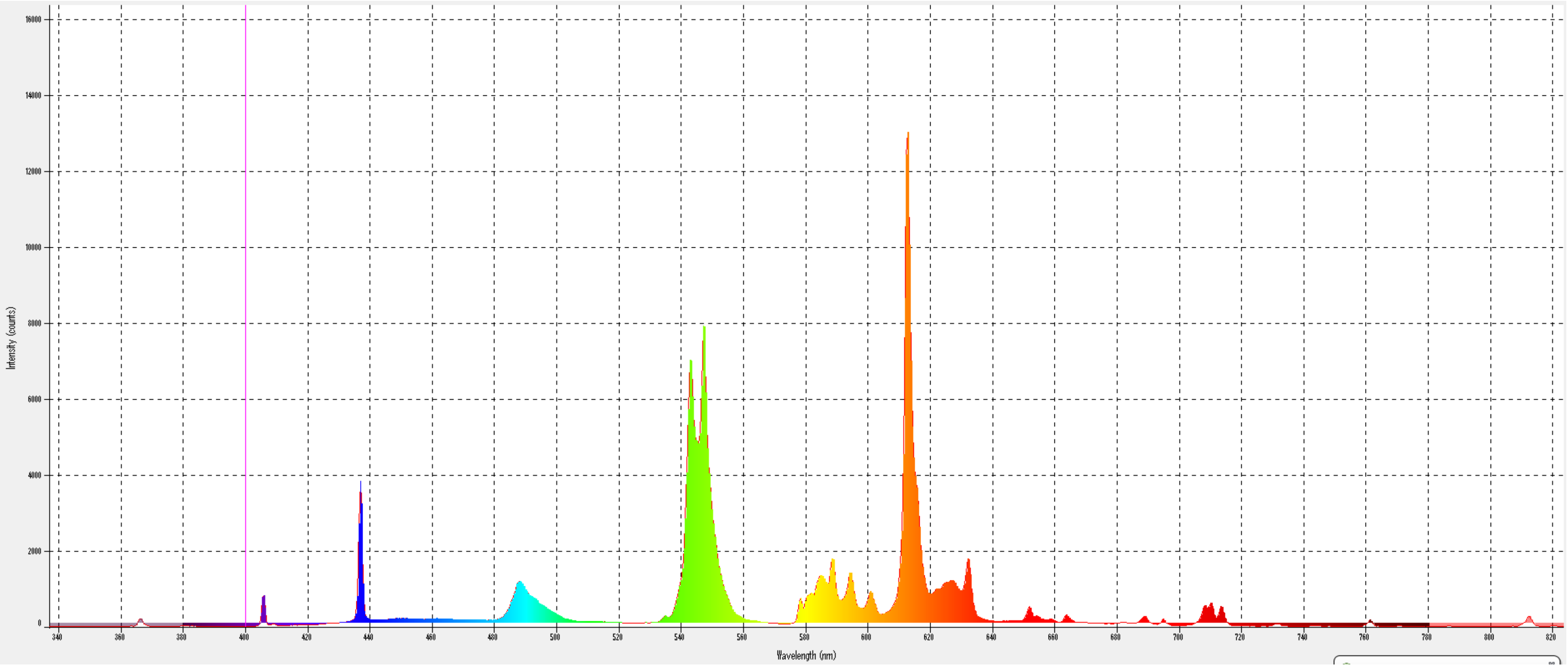

Figure S2: Spectrum of lights in a climate chamber Percival AR41-L2 (Percival Scientific) used in the experiment. The spectrum was measured by Spectrostopy education Kit (EDU-STS-VIS-PACK; Ocean Insight). Intensity of individual wavelengths is in relative units.
